# From Deep Hypothermia to Goal-Directed Perfusion: Contemporary Insights Into Perfusion Techniques for Pulmonary Endarterectomy, Including a Narrative Review and Perspectives

**DOI:** 10.7759/cureus.96478

**Published:** 2025-11-10

**Authors:** Youssef El Dsouki, Serdar Gunaydin, Sibel Aydin, Robert Kalimi, Michele Dell'Aquila, Ignazio Condello

**Affiliations:** 1 Perfusion, Sorbonne Université, Paris, FRA; 2 Cardiac Surgery, Ankara University School of Medicine, Ankara, TUR; 3 Perfusion, Koşuyolu High Specialization Education and Research Hospital, Istanbul, TUR; 4 Cardiothoracic Surgery, Northwell Health, Cardiovascular Institute, New York, USA; 5 School of Medicine and Surgery, University of Insubria, Varese, ITA

**Keywords:** cardiopulmonary bypass, ecmo, goal-directed perfusion, hypothermia, oxygen delivery, precision perfusion, pulmonary endarterectomy, temperature management

## Abstract

Pulmonary endarterectomy (PEA) remains the curative surgical option for chronic thromboembolic pulmonary hypertension (CTEPH), a condition requiring precise cardiopulmonary bypass (CPB) management to balance cerebral protection, myocardial preservation, and right-ventricular recovery. Historically based on deep hypothermic circulatory arrest (DHCA), PEA perfusion strategies have evolved toward moderate hypothermia, intermittent reperfusion, and goal-directed perfusion (GDP) guided by oxygen delivery (DO₂i) and metabolic indices. The advent of continuous perfusion monitoring, automated temperature regulation, and algorithm-driven management heralds a new era of precision perfusion in PEA. This narrative review and perspective integrates evidence from 24 peer-reviewed publications (2006-2025), including randomized controlled trials, multicenter observational studies, and technical reports. A systematic literature search was conducted in PubMed, Scopus, and Google Scholar using combinations of the keywords: pulmonary endarterectomy, cardiopulmonary bypass, goal-directed perfusion, oxygen delivery, hypothermia, and ECMO. Reference lists of eligible articles were cross-checked for additional sources. Inclusion criteria were: studies addressing CPB techniques or monitoring during PEA; data on temperature strategy, perfusion parameters, or metabolic guidance; and English-language full-text publications. Data were extracted and narratively synthesized to identify recurring patterns, innovations, and unmet needs in perfusion management. Findings confirmed the clinical equivalence between DHCA and antegrade cerebral perfusion in neurological outcomes and supported the safety of moderate hypothermia (20-24 °C) with intermittent reperfusion. Contemporary protocols adopt DO₂i targets ≥ 280-330 mL/min/m², integrating temperature-adjusted algorithms for individualized perfusion. Albumin-based prime and osmotic agents improve hemodynamic and cerebral stability, while Custodiol/HTK ensures sustained myocardial protection. ECMO is required in 7-10% of cases, predominantly for refractory right-ventricular dysfunction or reperfusion edema, with early initiation improving survival. The evolution of perfusion in PEA reflects a paradigm shift from empiric hypothermic models to dynamic, goal-directed, and technology-assisted perfusion. Real-time DO₂i monitoring, temperature-adjusted control, and algorithm-driven decision support are redefining intraoperative management. The next challenge lies in standardizing perfusion protocols and integrating automated systems capable of maintaining metabolic homeostasis throughout CPB. Collaborative multicenter studies and registries are warranted to validate these approaches and establish evidence-based guidelines for contemporary PEA perfusion.

## Introduction and background

**Figure 1:**
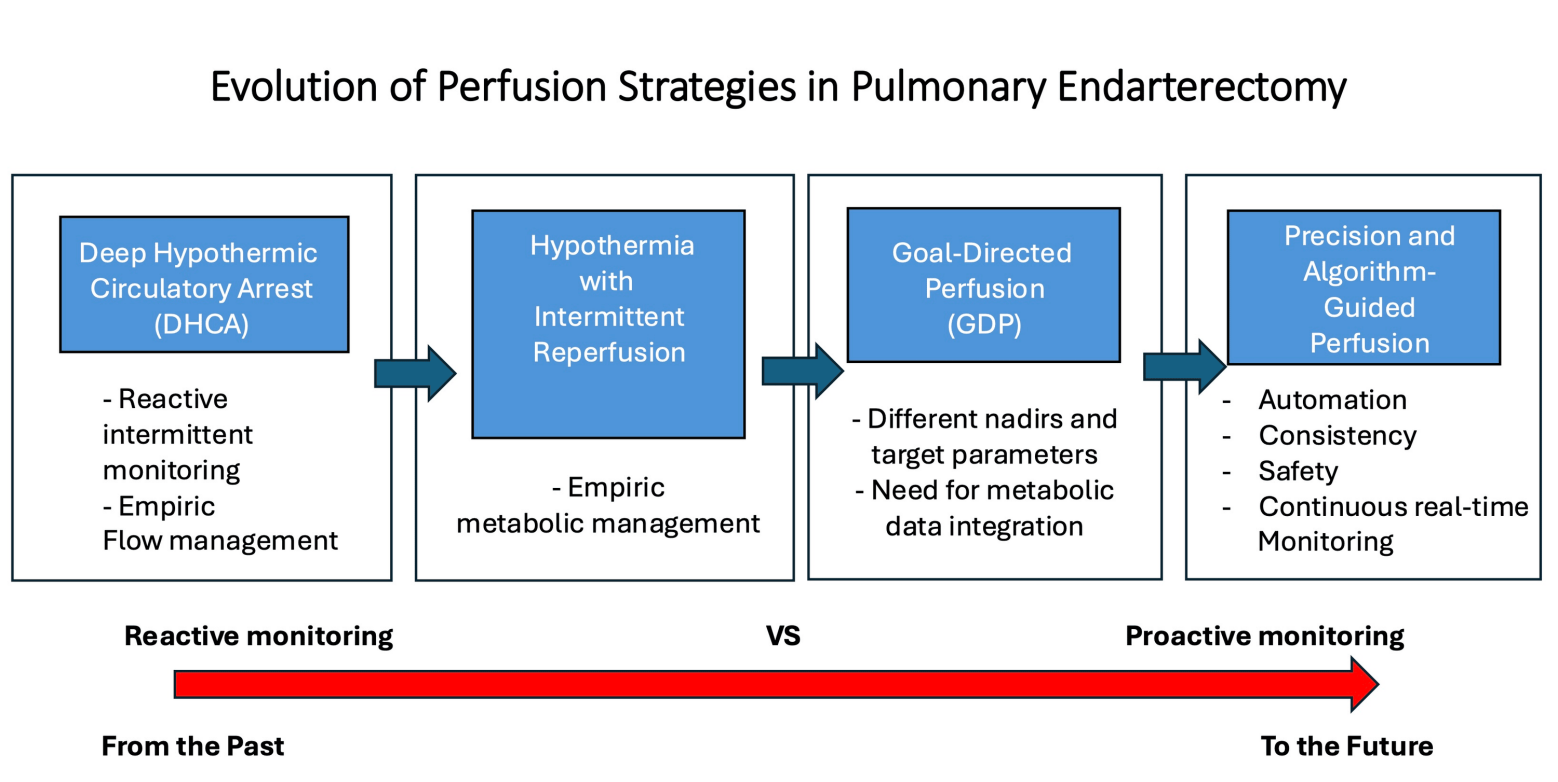
From hypothermia to goal-directed perfusion: evolution of perfusion strategies in pulmonary endarterectomy Visual summary of the transition from DHCA to MH, GDP, and the emerging concept of PP in PEA. Modern approaches integrate continuous DO₂i monitoring, temperature-adjusted control, and algorithm-guided feedback, marking the shift from reactive to proactive perfusion management. DHCA: Deep Hypothermic Circulatory Arrest; MH: Moderate Hypothermia; GDP: Goal-Directed Perfusion; PP: Precision Perfusion; PEA: Pulmonary Endarterectomy; DO₂i: Indexed Oxygen Delivery The figure is original.

Pulmonary endarterectomy (PEA) is the definitive and potentially curative surgical treatment for chronic thromboembolic pulmonary hypertension (CTEPH), a condition caused by organized thromboembolic obstruction of the pulmonary arteries leading to progressive right ventricular failure [1,2]. Since its first standardized execution at the University of California, San Diego, and subsequent refinements in European centers, such as Cambridge [3,4], PEA has evolved into one of the most complex procedures in cardiothoracic surgery, requiring close coordination among surgical, anesthetic, and perfusion teams. Historically, perfusion management during PEA relied on deep hypothermic circulatory arrest (DHCA) at 18-20 °C to provide a bloodless field and ensure cerebral protection [5,6]. This traditional approach, although effective, was guided by intermittent monitoring, mainly periodic blood gas analyses and flow-pressure parameters, so perfusion adjustments were reactive rather than preventive. Over the past two decades, accumulating evidence has questioned the need for profound hypothermia [7,8]. The PEACOG randomized trial demonstrated no neurological advantage of selective antegrade cerebral perfusion over DHCA [9], and subsequent studies confirmed that moderate hypothermia (20-24 °C) with intermittent reperfusion offers equivalent cerebral protection with shorter cardiopulmonary bypass (CPB) times [10,11]. These findings marked a gradual transition from empiric perfusion protocols toward goal-directed, physiology-based management in PEA. Conventional CPB monitoring provides only “snapshots” of rapidly changing physiology. In contrast, modern practice emphasizes continuous, data-driven perfusion guided by real-time assessment of oxygen delivery (DO₂), venous oxygen saturation (SvO₂), and carbon dioxide production (VCO₂) to anticipate and prevent metabolic imbalance [12-14]. Maintaining indexed oxygen delivery (DO₂i) ≥ 280-330 mL/min/m² has been associated with reduced postoperative organ dysfunction [15,16]. This proactive approach is particularly relevant in PEA, where prolonged CPB duration, deep cooling, and reperfusion injury increase metabolic demand. Recent developments, including temperature-adjusted DO₂ algorithms [17] and automated temperature control systems, have enabled individualized perfusion tailored to dynamic physiologic conditions. Improvements in circuit design and priming composition have enhanced biocompatibility and end-organ protection; for instance, 5% albumin-based priming solutions support better fluid balance and fewer postoperative complications [18,19]. Single-dose Custodiol/HTK cardioplegia remains a standard for myocardial protection during bilateral endarterectomy. Additionally, structured extracorporeal membrane oxygenation (ECMO) protocols are now part of PEA programs worldwide, supporting roughly 7-10% of patients with refractory right-ventricular failure or reperfusion lung injury [20,21]. Recent single-center and multicenter studies [12-15] have underscored the importance of protocol-driven perfusion and postoperative metabolic management. These innovations reflect a progressive shift from static, empiric approaches toward integrated and predictive perfusion models. Perfusion is increasingly recognized as a dynamic physiologic therapy that maintains systemic and cerebral homeostasis in real time. Within this context, PEA serves as a model for applying goal-directed and technology-assisted perfusion, where the integration of continuous monitoring, algorithmic guidance, and multidisciplinary collaboration may improve neurologic, renal, and hemodynamic outcomes [16]. Despite these advances, inter-institutional variability persists in perfusion and temperature strategies [13-17]. Moving from a reactive to a proactive paradigm requires not only new technologies but also a conceptual shift in the perfusionist’s role from technician to physiologic strategist [20,21]. This review summarizes current evidence and perspectives on continuous, goal-directed perfusion in PEA and discusses how evolving technologies may support more standardized and precision-driven cardiopulmonary management (Figure 1).

## Review

This work was conceived as a narrative review and perspective, designed to synthesize current evidence and highlight emerging concepts in perfusion management during PEA. Unlike systematic reviews, this methodology allows for a broader and more integrative appraisal of the literature, combining quantitative and qualitative findings with expert interpretation. Particular emphasis was placed on the evolution of cardiopulmonary bypass (CPB) strategies from DHCA to moderate hypothermia and goal-directed perfusion (GDP) and on the emerging role of continuous, proactive monitoring of oxygen delivery (DO₂i) and temperature-adjusted algorithms. A comprehensive literature search was conducted in PubMed, Scopus, and Google Scholar databases between January 2006 and August 2025. The following keywords and Boolean operators were used in different combinations: “pulmonary endarterectomy”, “cardiopulmonary bypass”, “chronic thromboembolic pulmonary hypertension”, “deep hypothermic circulatory arrest”, “goal-directed perfusion”, “oxygen delivery”, “temperature management”, and “ECMO”. Additional filters included studies published in English, involving human subjects, and available as full-text articles. Reference lists of key studies and reviews were also screened manually to identify additional relevant publications not captured by the initial search [4,10,11,13].

Publications were included if they met at least one of the following criteria: 1) focused on perfusion techniques, temperature strategies, or oxygen delivery monitoring during PEA; 2) reported clinical outcomes, safety data, or physiological endpoints related to CPB management in PEA [1,3,5,7,9,12,14,17]; 3) described innovations in goal-directed perfusion, DO₂ monitoring, or automated temperature algorithms applicable to PEA [16,18,21]; 4) provided multicenter, randomized, or prospective observational data relevant to metabolic or hemodynamic optimization during surgery.

Exclusion criteria included experimental or animal studies without human data, case reports lacking a perfusion-specific methodology, and non-peer-reviewed sources. All eligible studies were screened for relevance and methodological soundness. Extracted information included perfusion temperature and arrest strategy (DHCA, moderate hypothermia, or continuous perfusion); monitoring modalities (blood gas analysis, SvO₂, near-infrared spectroscopy, DO₂i); perfusion parameters (flow rates, hematocrit targets, pressure limits, prime composition); myocardial and neuroprotection strategies; and the use of ECMO in perioperative management [7,9]. The collected evidence was synthesized narratively and organized by thematic domains -temperature management, flow optimization, biochemical monitoring, and postoperative outcomes. Quantitative pooling or meta-analysis was not performed, as the included studies displayed marked heterogeneity in design, endpoints, and reporting, precluding statistical aggregation. The absence of formal quantitative synthesis is therefore justified by methodological variability and by the descriptive, perspective-based intent of this review. Although formal bias assessment tools (e.g., Preferred Reporting Items for Systematic reviews and Meta-Analyses (PRISMA), Risk Of Bias In Non-randomized Studies - of Interventions (ROBINS-I)) were not applied, the review adheres conceptually to PRISMA principles to ensure transparency, reproducibility, and structured reporting. Methodological rigor was evaluated qualitatively, considering study design, sample size, and clarity of perfusion protocol description. Priority was given to randomized trials [1], multicenter experiences [13], and contemporary technical reports on advanced perfusion monitoring and algorithmic control [16,18,20]. To complement the literature synthesis, this review integrates a perspective component, reflecting on the shift from reactive, intermittent monitoring to proactive, continuous management. This section draws on the most recent conceptual advances in temperature-adjusted oxygen delivery, automated perfusion control, and integrated DO₂i dashboards, representing the emerging frontier of precision perfusion in PEA [16,18-21].

Evolution of perfusion strategies in PEA

The evolution of perfusion strategies in pulmonary endarterectomy reflects a progressive refinement from empiricism to physiology-guided management. Initially, DHCA at 18-20 °C was universally adopted to provide a bloodless operative field and ensure cerebral protection [4]. This approach, pioneered in the early San Diego series, was later standardized in European centers such as Cambridge and Vienna [10,11]. However, the PEACOG (circulatory arrest versus cerebral perfusion during pulmonary endarterectomy surgery) randomized trial by Vuylsteke et al. demonstrated that selective antegrade cerebral perfusion offered no neurological advantage over conventional DHCA, suggesting that the depth of hypothermia could be moderated without compromising cerebral safety [1]. Subsequently, Mikus et al. and Lafci et al. reported favorable outcomes using moderate hypothermia (20-24 °C) with intermittent reperfusion, achieving comparable neurological protection and shorter CPB times [2,3]. These studies established the foundation for physiologically balanced perfusion, reducing ischemia-reperfusion injury and coagulopathy associated with profound cooling. More recently, Guan et al. and Liu et al. confirmed in multicenter analyses that tailored hypothermia (22-24 °C) preserves surgical visibility and cerebral safety while minimizing systemic inflammation [13,14].

A major evolution in PEA perfusion has been the adoption of GDP strategies aimed at maintaining adequate DO₂ and minimizing metabolic stress. Traditionally, perfusion flow and pressure were adjusted reactively, guided by intermittent blood gas analyses. The advent of continuous perfusion monitoring now enables real-time optimization of hemodynamics, hematocrit, and oxygen delivery before physiological deterioration occurs [16,18,19]. Salenger et al. demonstrated that maintaining DO₂i ≥ 280-330 mL/min/m² during CPB correlates with reduced postoperative organ dysfunction, particularly renal injury [16], while Mukaida et al. showed that cumulative oxygen delivery predicts acute kidney injury, underscoring the importance of sustained perfusion adequacy [21]. These findings are especially relevant in PEA, where prolonged CPB, low-flow intervals, and hypothermia challenge systemic oxygenation. The development of temperature-adjusted DO₂ algorithms further refined the GDP model by calibrating oxygen targets to metabolic demand across temperature ranges [18,19]. Moreover, El Dsouki and Condello demonstrated the feasibility of automated temperature and perfusion control, marking a step toward technology-assisted precision perfusion [20].

Optimal outcomes in PEA also depend on meticulous circuit priming and myocardial protection strategies. Furrer et al. showed that the inclusion of 5% albumin in the CPB prime improves colloid-oncotic stability, reduces fluid overload, and supports faster recovery [5]. Similarly, Edelman et al. confirmed the efficacy of Custodiol/HTK cardioplegia in providing prolonged myocardial protection for up to 120 minutes of cross-clamp time, essential in bilateral endarterectomies [6]. Recent technical reports emphasize the importance of aligning perfusion temperature and myocardial strategies to mitigate reperfusion injury, particularly in patients with right-ventricular dysfunction or comorbidities [12,14].

ECMO protocols have also become integral to PEA programs, supporting approximately 7-10% of patients with refractory right-ventricular failure or reperfusion lung injury [7-9]. Early work by Berman et al. established ECMO feasibility, while Sugiyama et al. and Bertazzo et al. expanded its indications to severe reperfusion injury [7-9]. Recent multicenter experiences confirm that early initiation and standardized ECMO protocols correlate with improved survival and reduced ventilator dependency [12-14]. Patel et al. further reported that intraoperative optimization of DO₂ may reduce postoperative ECMO requirements [17].

Recent contributions by El Dsouki and Condello and by Condello et al. illustrate how automated, temperature-adaptive algorithms can predict critical DO₂i thresholds and guide perfusion in real time [18-20]. Coupled with continuous metabolic and hemodynamic data, these systems promote a closed feedback perfusion model capable of anticipating physiological deterioration rather than reacting to it. This approach embodies the concept of precision perfusion, integrating analytics, dynamic flow control, and individualized temperature management. As emphasized by Furrer et al. and Salenger et al., applying these methods in PEA may help standardize outcomes and reduce inter-institutional variability [5,16]. In parallel with advances in temperature and perfusion management, recent attention has focused on cerebral oxygenation and depth of anesthesia monitoring, key factors in minimizing neurological complications during PEA. Continuous near-infrared spectroscopy (NIRS) enables real-time assessment of regional cerebral oxygen saturation (rSO₂), allowing timely adjustments of perfusion flow, hematocrit, and temperature within GDP protocols. Complementary monitoring through bispectral index (BIS) or processed EEG provides an additional safeguard by ensuring adequate depth of anesthesia and avoiding both over- and under-sedation during periods of circulatory arrest or low-flow perfusion. Recent evidence supports the integration of NIRS into GDP-based strategies for improved neurological protection. Sun and Wu (2024) demonstrated that maintaining cerebral oxygenation using NIRS monitoring significantly reduces postoperative delirium after cardiopulmonary bypass [22]. Similarly, Uysal et al. (2020) conducted a randomized controlled trial showing that optimized cerebral oxygenation correlates with better neurocognitive and perioperative outcomes in cardiac surgery [23]. Furthermore, Chiong et al. (2022) confirmed through a systematic review and meta-analysis that cerebral oximetry-guided management improves neurological outcomes and shortens hospital stay in patients undergoing cardiac surgery [24]. Together, these findings reinforce the importance of multimodal monitoring combining cerebral oximetry and anesthetic depth control as an integral component of precision perfusion. In the context of PEA, such strategies complement oxygen delivery-based management and help further reduce the risk of neurological complications, supporting a truly patient-specific approach to cardiopulmonary bypass (Table 1).

**Table 1:** Summary of key take-home messages, current evidence, and future perspectives in perfusion management for pulmonary endarterectomy

Topic / Domain	Current Evidence and Take-Home Messages	Future Perspectives and Research Directions	Key References
1. Temperature Management	Deep hypothermic circulatory arrest (DHCA) at 18–20 °C remains safe and effective but is increasingly replaced by moderate hypothermia (20–24 °C) with intermittent reperfusion, providing equivalent neurological protection and shorter bypass times.	Standardization of moderate hypothermia protocols and integration with goal-directed perfusion (GDP) targets to balance metabolic suppression and tissue oxygenation.	^(1–4, 13–15)^
2. Cerebral and Neurological Protection	The PEACOG trial demonstrated no superiority of antegrade cerebral perfusion over DHCA in neurological outcomes; moderate hypothermia yields similar neuroprotection when coupled with controlled reperfusion. Recent studies highlight the role of continuous cerebral oximetry (NIRS) and depth-of-anesthesia monitoring (BIS) in preventing postoperative delirium and cognitive dysfunction.	Development of hybrid perfusion strategies using continuous cerebral oximetry (NIRS) and oxygen delivery (DO₂)-guided flow control to further minimize neurological injury.	^(1–3, 16,22-24)^
3. Goal-Directed Perfusion (GDP)	Continuous monitoring of indexed oxygen delivery (DO₂i) ≥ 280–330 mL/min/m² reduces postoperative organ dysfunction and improves metabolic stability.	Incorporation of automated DO₂i algorithms, machine-learning–based prediction of perfusion adequacy, and integration with perfusion dashboards for real-time feedback.	^(16, 18–21)^
4. Reactive vs. Proactive Monitoring	Traditional CPB relies on intermittent blood gas analysis—reactive rather than preventive. Modern continuous monitoring (SvO₂, VCO₂, DO₂i) enables proactive management.	Implementation of fully integrated monitoring platforms with predictive analytics and early-warning alerts for hypoxia or perfusion mismatch.	^(16, 18–20)^
5. Prime Composition and Fluid Management	Albumin-enriched prime (5%) improves oncotic stability and reduces postoperative edema and hemodilution.	Comparative studies on colloid vs. crystalloid-based primes; individualized fluid management guided by dynamic hemodynamic indices.	^(5)^
6. Myocardial Protection	Custodiol/HTK provides reliable myocardial protection for up to 120 min of cross-clamp, supporting prolonged endarterectomy procedures.	Evaluation of metabolic cardioplegia and combined delivery methods (HTK + blood) tailored to PEA-specific ischemic profiles.	^(6, 12, 14)^
7. ECMO and Postoperative Support	ECMO is required in 7–10% of PEA cases, primarily for right-ventricular failure or reperfusion lung injury; early initiation improves survival.	Early prediction of ECMO requirement through intraoperative metabolic and hemodynamic indices; standardization of ECMO weaning criteria.	^(7–9, 12–14, 17)^
8. Endothelial and Temperature Interplay	The interaction between temperature, vascular resistance, and endothelial tone influences perfusion efficiency and systemic inflammation.	Research into endothelial biomarkers and temperature-responsive perfusion algorithms for personalized CPB modulation.	^(19)^
9. International and Institutional Variability	Significant inter-center heterogeneity persists in CPB management (temperature, flow targets, DO₂i use).	Creation of multicenter registries and consensus guidelines for standardized perfusion strategies in PEA.	^(13–15, 17)^
10. The New Paradigm: Precision Perfusion	Modern perfusion has transitioned from a static, reactive system to a dynamic, goal-directed, and data-driven practice.	Integration of continuous DO₂ monitoring, temperature-adjusted control, and AI-based automation to achieve full precision perfusion in PEA.	^(16, 18–21)^

## Conclusions

Perfusion management in pulmonary endarterectomy (PEA) is evolving from empiric, hypothermia-based models toward goal-directed and physiology-guided strategies. This transition reflects a shift from reactive correction after imbalance to proactive prevention of metabolic and hemodynamic instability. Continuous monitoring of oxygen delivery, temperature-adjusted algorithms, and data-driven flow control now support more consistent organ protection and intraoperative stability. Looking forward, the integration of automated control systems and predictive analytics may enhance standardization and reduce inter-institutional variability. However, achieving precision perfusion extends beyond technology; it requires a conceptual and cultural change, redefining the perfusionist’s role as an active partner in physiological management rather than a reactive technician. Ultimately, the pursuit of precision perfusion aims to make cardiopulmonary bypass during PEA safer, more consistent, and patient-specific, bridging innovation with evidence-based practice.
